# Too much is too little: Estimating the optimal physical activity level for a healthy mental state

**DOI:** 10.3389/fpsyg.2022.1044988

**Published:** 2023-01-13

**Authors:** Akiyoshi Shimura, Jiro Masuya, Katsunori Yokoi, Chihiro Morishita, Masayuki Kikkawa, Kazuki Nakajima, Chong Chen, Shin Nakagawa, Takeshi Inoue

**Affiliations:** ^1^Department of Psychiatry, Tokyo Medical University, Shinjuku-ku, Tokyo, Japan; ^2^School of Medicine, International University of Health and Welfare, Chiba, Japan; ^3^Division of Neuropsychiatry, Department of Neuroscience, Yamaguchi University Graduate School of Medicine, Ube, Yamaguchi, Japan

**Keywords:** physical activity, exercise, mental health, optimal level, depression, anxiety, resilience, insomnia vulnerability

## Abstract

**Introduction:**

Although physical activity and exercise are generally thought to have favorable effects on mental health, excessive physical activity may have unfavorable effects. In this study, the associations between physical activity and the states of mental health with U-shaped dose–response curves were hypothesized, and the ranges of physical activity resulting in optimal effects on mental health were investigated.

**Methods:**

A cross-sectional survey was conducted on 1,237 adult volunteers in 2017 and 2018. Of these volunteers, 526 participants validly answered the self-administered questionnaires asking about physical activity, depression, anxiety, resilience, insomnia vulnerability, and life events. A comparison of mental health measures by physical activity levels and quadratic equation model regressions were performed.

**Results:**

No significant linear associations between physical activity levels and mental health measurements were observed; however, the U-shaped, quadratic equation models indicated a significance. The following levels of physical activity per week optimized the mental health measurements values of the participants: 6,953 MET-minutes and 25.70 h for depression, 5,277 MET-minutes and 21.60 h for state anxiety, 5,678 MET-minutes and 22.58 h for trait anxiety, 25.41 h for resilience, and 9,152 MET-minutes and 31.17 h for insomnia vulnerability.

**Conclusion:**

Physical activities in the optimal range were associated with more favorable mental health measurements. Physical activities that were too much or too long and outside of the optimal range were associated with less favorable mental health measurements.

## Introduction

Physical activity and exercise generally result in favorable effects on mental health. The association between physical activity and depression, anxiety, neuroticism, resilience, and insomnia were indicated in various studies. Meta-analyses have indicated the treatment effects of physical activity and exercise on the symptoms of people with depression (Krogh et al., 2010; Bridle et al., 2012; Josefsson et al., 2014; Schuch et al., 2016; Nebiker et al., 2018), and exercise is expected to decrease and prevent depressive symptoms not only in patients but also in the general population (Brown et al., 2005; Teychenne et al., 2008; de Zeeuw et al., 2010; Chekroud et al., 2018). Regarding other mental health outcomes, physical activity also decreases the anxiety levels of patients (Petruzzello et al., 1991; Hansen et al., 2001; Herring et al., 2010; Jayakody et al., 2014; Wegner et al., 2014; Marcos de Souza Moura et al., 2015; Stonerock et al., 2015; Aylett et al., 2018), is associated with increased resilience (Childs and de Wit, 2014), and also reduces insomnia symptoms (Driver and Taylor, 2000; Baron et al., 2013; Kredlow et al., 2015; Kelley and Kelley, 2017; Banno et al., 2018) and may also prevent insomnia (Morse et al., 2019).

There are multiple possible biological mechanisms by which physical activity has positive effects on mental health. For example, exercise improves brain-derived neurotrophic factor (BDNF) secretion levels in humans (Szuhany et al., 2015), and modulates cortical glutamate and γ-amino butyric acid levels (Maddock et al., 2016). Physical activity augments neurogenesis and synaptogenesis in the hippocampus, and also increases serotonin levels in animals (Kondo and Shimada, 2015). These biological changes may decrease the level of depression, anxiety, or insomnia in humans.

On the other hand, the dose–response association between the level of physical activity and mental health remains unknown. A previous large-scale cross-sectional study (Hamer et al., 2009) and a randomized control trial (Dunn et al., 2005) indicated a dose–response association of relatively low level physical activity with psychological distress and depression; however, a review concluded that there was little evidence of a dose–response association of physical activity with depression and anxiety (Dunn et al., 2001). Particularly regarding relatively high levels of physical activity, it remains unclear whether a higher level of physical activity is simply better or not. Regarding physical health, physical activities that are too vigorous or too long will cause physical and functional impairments in individuals, which is known as “overtraining syndrome” (Johnson and Thiese, 1992; Armstrong and VanHeest, 2002; Wyatt et al., 2013). This adverse effect of too much physical activity is not only limited to physical health but is expected to also affect mental health (Johnson and Thiese, 1992). A possible path by which overexercising may lead to depressive or anxiety symptoms is through the hypothalamic–pituitary–adrenal axis, because overexercising causes the secretion of cytokines and cortisol (Smith, 2000; Wyatt et al., 2013; Anderson et al., 2016). This suggests that there is a nonlinear association between the amount of physical activity and mental health.

If both too little and too much physical activity have unfavorable effects on mental health, a nonlinear dose–response association, such as a U-shaped curve might be observed. In fact, a previous large-scale study (Chekroud et al., 2018) suggested a potential L-shaped or U-shaped association between physical activity level and mental health, and that performing certain types of exercise on most days of the month leads to unfavorable effects on mental health. However, to date, there have been no studies analyzing whether the model of the U-shaped association is true or not, and moreover, no study has clarified the optimal level of physical activity for mental health.

Here, we performed a cross-sectional study to analyze the associations between the amount of physical activity and representative mental health components, such as depression, anxiety, neuroticism, resilience, and insomnia, by assessing the quadratic equation model to confirm the hypothesis that there are significant non-linear, U-shaped dose–response associations between physical activity and aspects of mental health.

## Materials and methods

### Study design and participants

This study was designed as a cross-sectional survey using self-administered questionnaires. The data of the participants have been used in previous studies investigating the associations between job stress and sleep rhythm (Miyama et al., 2020) or resilience (Sameshima et al., 2020). First, the effect size of physical activity on mental health was conservatively estimated as “small to medium” (Cohen’s *d* = 0.2 to 0.5). Thus, the optimal sample size was calculated to be 105 to 651, and hence approximately 1,300 participants were recruited, with an expected valid response rate of 50%. Subsequently, a paper-based survey form was distributed to 1,237 Japanese volunteers from the nonclinical adult population, from September 2017 to May 2018. The volunteers were nonclinical workers who were recruited by convenience sampling through person-to-person acquaintances at a University located in Tokyo, which is an urban area of Japan. Of those, 526 volunteers (42.5%) gave informed consent and valid answers to the questionnaire without any missing data. Answers were collected anonymously. There were no special inclusion or exclusion criteria. The study was conducted in accordance with the Declaration of Helsinki (amended in Fortaleza in 2013) and approved by the Tokyo Medical University Research Ethics Committee (study approval no.: SH3502).

### Questionnaires

The International Physical Activity Questionnaire (IPAQ) was used to assess the physical activity level of the participants. For assessment of the mental health states of the participants, The Patient Health Questionnaire-9 (PHQ-9) was used for depression, The State–Trait Anxiety Inventory, Form Y (STAI-Y) for anxiety, The Connor-Davidson Resilience Scale (CD-RISC) for resilience, and The Ford Insomnia Response to Stress Test (FIRST) for insomnia vulnerability.

IPAQ is a self-administered questionnaire that is widely used all over the world (Craig et al., 2003), and the validated Japanese short-form version (Murase, 2002) was used in this study. IPAQ assesses physical activity in the following 4 domains: leisure-time physical activity, domestic and gardening activities, work-associated physical activity, and transport-associated physical activity, and the short form asks about the following 3 specific types of activity: walking, moderate-intensity activities, and vigorous-intensity activities. IPAQ enables estimation of the total physical activity intensity indicated as METs and total physical activity duration. One MET is equivalent to the resting metabolic kilocalories for a person per bodyweight in kg. For example, if a person with a 50 kg bodyweight walks, which has a 3 METs score, for 60 min, the metabolic kilocalories are estimated as 50 kg * 3 METs * 1 h = 150 kcal, and is described as 3 METs * 60 min = 180 MET-minutes.

PHQ-9 (Kroenke et al., 2001) and its validated Japanese version (Muramatsu et al., 2007) was used for assessing depressive symptoms. PHQ-9 is a self-administered questionnaire consisting of 9 items associated with depression. PHQ-9 has high sensitivity and specificity for clinical major depressive episodes, with higher scores indicating a severer depressive state.

STAI-Y (Spielberger et al., 1971) and its validated Japanese version (Hidano et al., 2000) was used to assess trait and transient state anxiety. STAI-Y consists of a 40-item self-report questionnaire and subjects answer how they feel anxiety generally or at a particular moment specified in each item. In both the trait anxiety category and state anxiety category, higher scores indicate higher levels of anxiety.

CD-RISC (Connor and Davidson, 2003) and its validated Japanese version (Nishi et al., 2010) was used to assess psychological resilience. CD-RISC is a 25-item Likert scale questionnaire that assesses the “personal qualities that enable one to thrive in the face of adversity.” A higher score on CD-RICS indicates that the person is in a more psychologically resilient state.

FIRST (Drake et al., 2004) and its validated Japanese version (Nakajima et al., 2014) was used to assess the tendency of having insomnia owing to stressful conditions. FIRST consists of a 9-item self-report questionnaire that analyzes the level of trait vulnerability and sleep reactivity to experience situational insomnia. Persons with higher scores on FIRST are more vulnerable to and easily develop insomnia as a result of their environment.

To assess the effects of private events on mental health measures, the validated Japanese version (Nakai et al., 2014) of Life Experiences Survey (LES; Sarason et al., 1978) was used. LES is a 57-item self-report measure assessing major life events in the past 12 months. Each item is given a score ranging from −3 to +3. LES positive change scores sum the impact ratings of positive life events, and LES negative change scores sum the impact ratings of negative life events.

### Data analysis and quadratic equation model

**Figure 1:**
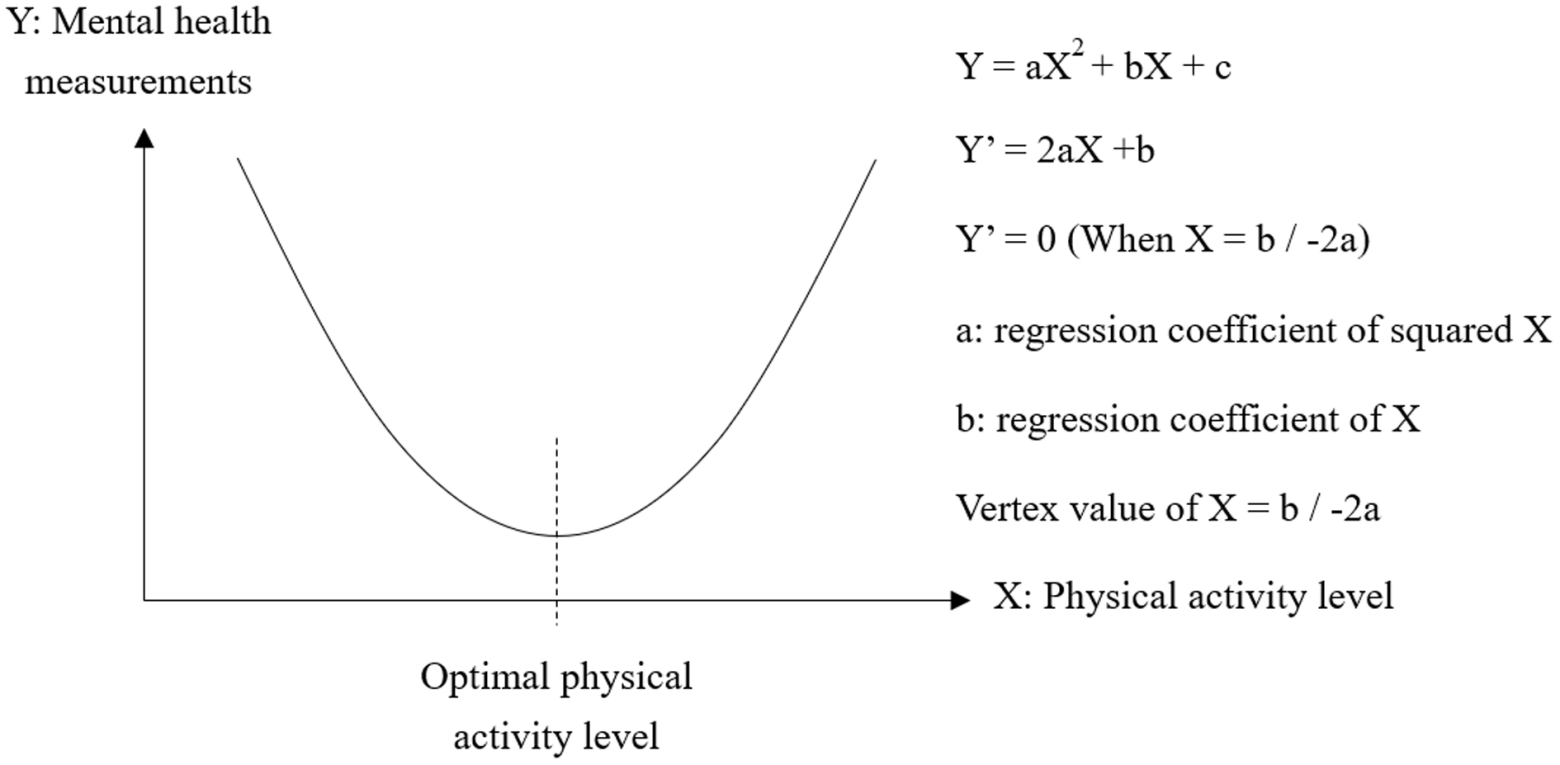
Explanation of quadratic equation model regression analysis.

First, the association between physical activities and mental health measurements were investigated, and a comparison by demographic variables was performed. Second, linear regression analyses were performed using total weekly physical activity volume and duration as independent variables, and mental health measurements as dependent variables. For the linear regression analyses, the squared term of total physical activity volume and duration were added. This enabled analysis of the significance of the U-shaped curve hypothesis, and estimation of physical activity volume and duration values that minimize or maximize the mental health measurements if it is significant, similar to the method that calculating the optimal BMI 22 for minimizing morbidity (Tokunaga et al., 1991; Figure 1).

**Figure 2:**
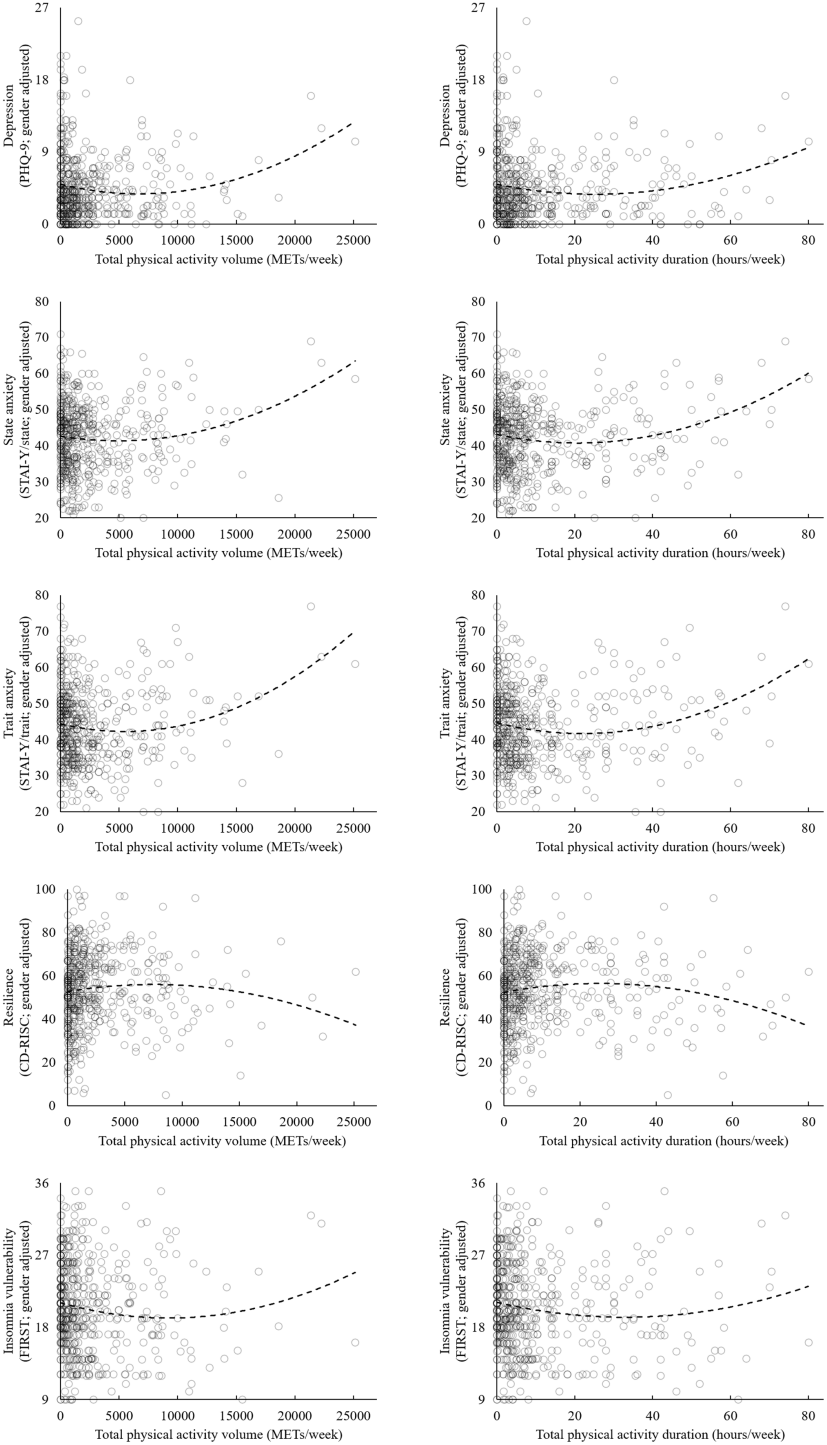
Physical activity levels and mental health measurements.

Statistical analyses were performed using IBM SPSS Statistics version 23 software. A value of *p* of less than 0.05 was considered to indicate a statistically significant difference between groups.

### Results

Participants comprised 228 men (43.3%) and 297 women (56.5%), and 1 other (0.2%). The mean age was 41.2 years [standard deviation (SD) = 11.9 years]. The mean weekly total physical activity volume was 2,479.6 MET-minutes (SD = 3,467.3 MET-minutes), which is equivalent to 2,479.6 kcal metabolism per week for participants with a 60-kg bodyweight. The mean weekly total physical activity duration was 10.4 h (SD = 14.3 h). The values of Cronbach’s α of the mental health measurements were PHQ9: 0.854, STAI-Y: 0.783, CD-RISC: 0.948, and FIRST: 0.895, with acceptable to high (Cortina, 1993) reliability. The results of the comparison of gender differences and Pearson’s correlation analysis are shown in Table 1. Significant gender differences were observed in mental health measurements (*p* < 0.05, *t*-test). Women had higher scores on depression, trait and state anxiety, and insomnia vulnerability, and a lower score on resilience.

**Table 1:** Demographic characteristics, physical activity, and mental health measurements of the participants.

Characteristic or life habit	N (%) or mean (SD)	Mean (SD) or Pearson’s correlation (*r*)						
		PA volume	PA duration	Depression	State anxiety	Trait anxiety	Resilience	Insomnia vulnerability
Demographic characteristic								
Male	228 (43.3%)	2,722.4 (3,317.6)	11.08 (13.00)	3.27 (3.79)**	39.8 (9.3)**	41.7 (10.2)*	56.9 (18.5)*	17.3 (5.6)**
Female	297 (56.5%)	2,292.1 (3,579.9)	9.93 (15.23)	4.51 (4.36)**	42.5 (9.9)**	43.7 (10.5)*	53.6 (16.2)*	20.5 (5.8)**
Age	41.2 (11.9)	−0.021	−0.024	−0.025	0.019	−0.052	0.006	0.082
LES: positive life experiences score	1.60 (2.69)	0.023	0.019	−0.106*	−0.125**	−0.135**	0.206**	−0.048
LES negative life experiences score	1.82 (3.32)	0.034	0.040	0.324**	0.298**	0.310**	−0.100*	0.221**
Physical activity per week								
Total physical activity volume (MET-minutes)	2,479.6 (3467.3)	1	0.968**	0.003	0.074	0.068	0.016	−0.074
Total physical activity duration (hours)	10.4 (14.3)	0.968**	1	−0.001	0.074	0.057	−0.003	−0.071
Mental health measurements								
Depression (PHQ-9)	3.97 (4.16)	0.003	−0.001	1	0.535**	0.642**	−0.385**	0.433**
State anxiety (STAI-Y/state)	41.3 (9.7)	0.074	0.074	0.535**	1	0.774**	−0.421**	0.411**
Trait anxiety (STAI-Y/trait)	42.9 (10.4)	0.068	0.057	0.642**	0.774**	1	−0.578**	0.489**
Resilience (CD-RISC)	55.0 (17.3)	0.016	−0.003	−0.385**	−0.421**	−0.578**	1	−0.309**
Insomnia vulnerability (FIRST)	19.1 (5.9)	−0.074	−0.071	0.433**	0.411**	0.489**	−0.309**	1

PA, physical activity; PHQ-9, Patient Health Questionnaire-9; STAI-Y, State–Trait Anxiety Inventory, Form Y; CD-RISC, Connor-Davidson Resilience Scale; FIRST, Ford Insomnia Response to Stress Test; LES, Life Experiences Survey. *p < 0.05, **p < 0.01 (Student t-test or Pearson’s correlation).

Regression analyses were performed to investigate the associations between physical activity levels and mental health measurements. Univariate linear regression analyses demonstrated that there were no linear associations between physical activity level (both volume and duration), and mental health measurements. However, the quadratic equation models indicated statistical significances. Table 2 shows the result of the regression analyses of the effects of total physical activity volume on mental health measures. For depression, 6,953 MET-minutes per week minimized the PHQ-9 value (*F* = 11.632, *p* < 0.001); for state anxiety, 5,277 MET-minutes per week minimized the value of STAI-Y/state (*F* = 7.441, *p* < 0.001); for trait anxiety, 5,678 MET-minutes per week minimized the value of STAI-Y/trait (*F* = 9.773, *p* < 0.001); and for insomnia vulnerability, 9,152 MET-minutes per week minimized the value of FIRST (*F* = 5.350, *p* = 0.005). Regarding resilience, although the physical activity volume model did not show statistical significance (*F* = 2.383, *p* = 0.093), after adjusting for gender, 7,468 MET-minutes per week maximized the value of CD-RISC score (*F* = 2.619, *p* = 0.050). The results adjusted by gender and positive/negative life experiences score indicated similar values. Supplementary Table 1 shows the results divided by gender. Each optimal physical activity level among males was higher than that of females. Table 3 shows the result of the regression analyses of the effects of total physical activity duration on mental health measures. For depression, 25.70 h per week minimized the value of PHQ-9 (*F* = 6.906, *p* = 0.001); for state anxiety, 21.60 h per week minimized the value of STAI-Y/state (*F* = 10.748, p < 0.001); for trait anxiety, 22.58 h per week minimized the value of STAI-Y/trait (*F* = 10.198, *p* < 0.001); for resilience, 25.41 h per week maximized the value of CD-RISC (*F* = 4.031, *p* = 0.018); and for insomnia vulnerability, 31.17 h per week minimized the value of FIRST (*F* = 5.726, *p* = 0.003). The results adjusted by gender and positive/negative life experiences score indicated similar values. Supplementary Table 2 shows the results divided by gender. Each optimal physical activity duration among males was longer than that of females. The calculated graphs of the U-shape curve were shown in Figure 2.

**Table 2:** Regression analyses of the effect of physical activity volume on mental health measures.

Explanatory variable	Univariate model		Quadratic equation model		Quadratic equation model: gender adjusted		Quadratic equation model: gender and LES adjusted	
	Coefficient	value of *p*	Coefficient	value of *p*	Coefficient	value of *p*	Coefficient	value of *p*
For depression (PHQ-9)								
PA volume (MET-minutes/week)	3.991 × 10^−6^	0.940	−414.882 × 10^−6^	<0.001	−366.946 × 10^−6^	0.002	−377.570 × 10^−6^	<0.001
(PA volume)^2^	–		29.833 × 10^−9^	<0.001	27.164 × 10^−9^	<0.001	26.971 × 10^−9^	<0.001
Gender (female vs. male)	1.309	<0.001			1.161	0.002	0.819	0.019
LES: positive life experiences							−0.178	0.006
LES: negative life experiences score							0.401	<0.001
*F*-value	0.006	0.940	11.632	<0.001	8.827	<0.001	19.186	<0.001
Adjusted *R*^2^	−0.002		0.028		0.044		0.151	
Vertex value of PA (MET-minutes/week)			6,953		6,754		6,999	
For state anxiety (STAI-Y/state)								
PA volume (MET-minutes/week)	208.864 × 10^−6^	0.092	−632.241 × 10^−6^	0.021	−536.634 × 10^−6^	0.049	−538.941 × 10^−6^	0.037
(PA volume)^2^	–		59.906 × 10^−9^	<0.001	54.729 × 10^−9^	0.002	53.201 × 10^−9^	0.001
Gender (female vs. male)	2.583	0.003			2.371	0.006	1.674	0.041
LES: positive life experiences							−0.506	<0.001
LES: negative life experiences score							0.880	<0.001
*F*-value	2.850	0.092	7.441	<0.001	7.690	<0.001	17.641	<0.001
Adjusted *R*^2^	0.004		0.024		0.038		0.140	
Vertex value of PA (MET-minutes/week)			5,277		4,902		5,065	
For trait anxiety (STAI-Y/trait)								
PA volume (MET-minutes/week)	206.547 × 10^−6^	0.123	−873.636 × 10^−6^	0.003	−803.979 × 10^−6^	0.007	−810.625 × 10^−6^	0.004
(PA volume)^2^	–		76.934 × 10^−9^	<0.001	73.139 × 10^−9^	<0.001	71.557 × 10^−9^	<0.001
Gender (female vs. male)	2.040	0.029			1.719	0.064	0.898	0.307
LES: positive life experiences							−0.580	<0.001
LES: negative life experiences score							0.998	<0.001
*F*-value	2.389	0.123	9.773	<0.001	7.743	<0.001	19.177	<0.001
Adjusted *R*^2^	0.003		0.033		0.038		0.151	
Vertex value of PA (MET-minutes/week)			5,678		5,496		5,664	
For resilience (CD-RISC)								
PA volume (MET-minutes/week)	78.963 × 10^−6^	0.721	1,022.168 × 10^−6^	0.038	909.725 × 10^−6^	0.066	811.128 × 10^−6^	0.006
(PA volume)^2^	–		−67.178 × 10^−9^	0.032	−60.910 × 10^−9^	0.053	−53.712 × 10^−9^	0.024
Gender (female vs. male)	−3.090	0.046			−2.720	0.080	−2.209	<0.001
LES: positive life experiences							1.339	0.281
LES: negative life experiences score							−0.538	<0.001
*F*-value	0.127	0.721	2.383	0.093	2.618	0.050	7.066	<0.001
Adjusted *R*^2^	−0.002		0.005		0.015		0.056	
Vertex value of PA (MET-minutes/week)			ns		7,468		7,551	
For insomnia vulnerability (FIRST)								
PA volume (MET-minutes/week)	−127.124 × 10^−6^	0.094	−545.686 × 10^−6^	0.001	−421.118 × 10^−6^	0.010	−441.135 × 10^−6^	0.006
(PA volume)^2^	–		29.811 × 10^−9^	0.005	22.760 × 10^−9^	0.029	23.139 × 10^−9^	0.024
Gender (female vs. male)	3.145	<0.001			2.972	<0.001	2.638	<0.001
LES: positive life experiences							−0.101	0.281
LES: negative life experiences score							0.362	<0.001
*F*-value	2.817	0.094	5.350	0.005	14.917	<0.001	14.025	<0.001
Adjusted *R*^2^	0.004		0.017		0.075		0.113	
Vertex value of PA (MET-minutes/week)			9,152		9,251		9,532	

PA, physical activity; PHQ-9, Patient Health Questionnaire-9; STAI-Y, State–Trait Anxiety Inventory, Form Y; CD-RISC, Connor-Davidson Resilience Scale; FIRST, Ford Insomnia Response to Stress Test; LES, Life Experiences Survey.

**Table 3:** Regression analyses of the effects of physical activity duration on mental health measures.

Explanatory variable	Univariate model		Quadratic equation model		Quadratic equation model: gender adjusted		Quadratic equation model: gender and LES adjusted	
	Coefficient	value of *p*	Coefficient	value of *p*	Coefficient	value of *p*	Coefficient	value of *p*
For depression (PHQ-9)								
PA duration (hours/week)	−0.244 × 10^−3^	0.985	−119.965 × 10^−3^	<0.001	−102.728 × 10^−3^	0.003	−110.039 × 10^−3^	<0.001
(PA duration)^2^	–		2.334 × 10^−3^	<0.001	2.028 × 10^−3^	0.001	2.099 × 10^−3^	<0.001
Gender (female vs. male)	1.309	<0.001	–		1.040	0.005	0.707	0.042
LES: positive life experiences							−0.169	0.008
LES: negative life experiences score							0.405	<0.001
*F*-value	0.000	0.985	6.906	0.001	7.361	<0.001	18.448	<0.001
Adjusted *R*^2^	−0.002		0.028		0.038		0.143	
Vertex value of PA (hours/week)			25.70		25.32		26.21	
For state anxiety (STAI-Y/state)								
PA duration (hours/week)	50.600 × 10^−3^	0.092	−270.378 × 10^−3^	0.021	−233.669 × 10^−3^	0.004	−242.768 × 10^−3^	0.002
(PA duration)^2^	–		6.258 × 10^−3^	<0.001	5.620 × 10^−3^	<0.001	5.666 × 10^−3^	<0.001
Gender (female vs. male)	2.583	0.003	–		2.181	0.010	1.523	0.060
LES: positive life experiences score							−0.481	0.001
LES: negative life experiences score							0.873	<0.001
*F*-value	2.922	0.088	10.748	<0.001	9.549	<0.001	18.806	<0.001
Adjusted *R*^2^	0.004		0.036		0.047		0.145	
Vertex value of PA (hours/week)			21.60		20.79		21.42	
For trait anxiety (STAI-Y/trait)								
PA duration (hours/week)	41.523 × 10^−3^	0.193	−305.694 × 10^−3^	<0.001	−280.535 × 10^−3^	0.001	−292.496 × 10^−3^	<0.001
(PA duration)^2^	–		6.770 × 10^−3^	<0.001	6.331 × 10^−3^	0.000	6.405 × 10^−3^	<0.001
Gender (female vs. male)	2.040	0.029	–		1.499	0.103	0.716	0.441
LES: positive life experiences score							−0.541	<0.001
LES: negative life experiences score							0.997	<0.001
*F*-value	1.696	0.193	10.198	<0.001	7.736	<0.001	19.119	<0.001
Adjusted *R*^2^	0.001		0.034		0.037		0.148	
Vertex value of PA (hours/week)			22.58		22.16		22.83	
For resilience (CD-RISC)								
PA duration (hours/week)	3.479 × 10^−3^	0.947	378.017 × 10^−3^	0.009	334.418 × 10^−3^	0.023	305.898 × 10^−3^	0.033
(PA duration)^2^	–		−7.438 × 10^−3^	0.005	−6.663 × 10^−3^	0.012	−6.068 × 10^−3^	0.020
Gender (female vs. male)	−3.090	0.046	–		−2.634	0.086	−2.207	0.144
LES: positive life experiences score							1.271	<0.001
LES: negative life experiences score							−0.544	0.015
*F*-value	0.004	0.947	4.031	0.018	3.678	0.012	7.517	<0.001
Adjusted R^2^	−0.002		0.011		0.015		0.060	
Vertex value of PA (hours/week)			25.41		25.09		25.21	
For insomnia vulnerability (FIRST)								
PA duration (hours/week)	−29.352 × 10^−3^	0.094	−165.573 × 10^−3^	0.001	−116.540 × 10^−3^	0.017	−126.040 × 10^−3^	0.090
(PA duration)^2^	–		2.660 × 10^−3^	0.003	1.775 × 10^−3^	0.045	−1.886 × 10^−3^	0.030
Gender (female vs. male)	3.145	<0.001	–		−2.634	<0.001	2.666	<0.001
LES: positive life experiences score							−0.097	0.291
LES: negative life experiences score							0.358	<0.001
*F*-value	2.639	0.105	5.726	0.003	15.570	<0.001	14.415	<0.001
Adjusted *R*^2^	0.003		0.018		0.077		0.114	
Vertex value of PA (hours/week)			31.17		32.84		33.42	

PA, physical activity; PHQ-9, Patient Health Questionnaire-9; STAI-Y, State–Trait Anxiety Inventory, Form Y; CD-RISC, Connor-Davidson Resilience Scale; FIRST, Ford Insomnia Response to Stress Test; LES, Life Experiences Survey.

These results indicated that physical activity levels of approximately +1 SD can be regarded as optimal physical activity levels in this population. To compare between people performing different levels of physical activity, participants were divided into 3 groups; i.e., lower-intermediate (performing physical activities of less than the average volume or duration), higher-intermediate (performing physical activities of more than the average volume or duration and less than +1 SD), and high (more than +1 SD); the mental health measurements of each group are shown in Table 4. One-way ANOVA indicated that there are group differences, and the higher-intermediate group tended to show more favorable mental health measurement scores than the other groups.

**Table 4:** Comparison between physical activity level and mental health measurements.

Physical activity level	N (%)	Mean (SD)						
		PA volume (MET-minutes/week)	PA duration (hours/week)	Depression	State anxiety	Trait anxiety	Resilience	Insomnia vulnerability
Physical activity volume per week								
Lower-intermediate (<mean)	368 (71.6%)	848.8 (832.2)	3.35 (2.91)	4.07 (4.33)	41.3 (9.8)	42.8 (10.4)	54.8 (17.4)	19.4 (5.8)
Higher-intermediate (≥mean, <1 SD)	71 (13.8%)	3816.2 (1354.1)	15.84 (4.04)	2.99 (2.83)	39.3 (7.9)	40.2 (8.6)	59.6 (15.2)	18.1 (5.8)
High (≥1 SD)	75 (14.6%)	9,215.6 (4152.8)	40.44 (13.32)	4.42 (4.24)	43.6 (10.6)	45.6 (11.8)	52.1 (17.7)	18.8 (6.5)
*F*-value		694.474***	1,326.618***	2.583	3.762*	5.051**	3.735*	1.564
Physical activity duration per week								
Lower-intermediate (<mean)	363 (70.6%)	792.2 (719.3)	3.37 (3.21)	4.09 (4.41)	41.3 (9.7)	43.2 (10.3)	54.2 (17.5)	19.5 (5.9)
Higher-intermediate (≥mean, <1 SD)	87 (16.9%)	3962.1 (1100.8)	16.72 (6.39)	3.52 (3.08)	38.4 (8.7)	39.1 (9.2)	60.2 (15.9)	17.5 (5.4)
High (≥1 SD)	64 (12.5%)	1,0034.5 (3,980.5)	42.06 (13.63)	4.11 (4.10)	44.1 (10.2)	46.1 (11.8)	53.2 (17.7)	18.5 (6.5)
*F*-value		966.294***	1,154.437***	0.677	6.762**	9.070***	4.602**	4.475*

PA, physical activity; PHQ-9, Patient Health Questionnaire-9; STAI-Y, State–Trait Anxiety Inventory, Form Y; CD-RISC, Connor-Davidson Resilience Scale; FIRST, Ford Insomnia Response to Stress Test. *p < 0.05, **p < 0.01, ***p < 0.001 (one-way ANOVA).

### Discussion

In this study, we statistically confirmed the nonlinear, but U-shaped associations between physical activity level and various mental health measurements. Approximately 21 to 31 h of physical activity per week (3 to 4.5 h daily), or 5.3 to 9.2 k METs-minutes (750 kcal to 1,300 kcal daily for a 60-kg person) was found to be associated with an optimal mental health state, regarding depression, anxiety, resilience, and insomnia. Under regular circumstances, exercise is expected to have favorable effects on mental health; however, beyond the optimal range, i.e., too intense or too long physical activity, is associated with impairment of mental health.

To date, no guidelines or optimal targets of exercise or physical activity level to efficiently maintain mental health have been established. The findings in this study indicating that a target level of physical activity to obtain an optimal mental health state can be set would be beneficial in clinical scenes or for mass-intervention from the aspect of public health. As described in previous studies, the associations between physical activity level and depression (Brown et al., 2005; Teychenne et al., 2008; de Zeeuw et al., 2010; Chekroud et al., 2018), anxiety (Petruzzello et al., 1991; Hansen et al., 2001; Herring et al., 2010; Jayakody et al., 2014; Wegner et al., 2014; Marcos de Souza Moura et al., 2015; Stonerock et al., 2015; Aylett et al., 2018), resilience (Childs and de Wit, 2014), and insomnia vulnerability (Morse et al., 2019) were also confirmed in this study. Particularly, in the “not too much” range, physical activity was associated linearly with mental health measurements. Regarding depression, although a review has concluded that both shorter and longer durations and vigorous-intensity physical activities are effective in reducing the likelihood of depression (Teychenne et al., 2008), the definitions of “longer” and “vigorous” were not specified, and a large-scale study (Chekroud et al., 2018) indicated that exercising on most days of the month results in unfavorable effects on mental health. The lack of a threshold regarding “how much is too much” is a common problem in mental health, such as regarding depression, and the findings in this study may contribute toward setting the standard for further studies.

This study has several limitations. First, this study had a cross-sectional design, and hence the causal associations are unclear and cannot be determined. There is also the possibility that too much physical activity did not cause mental health impairments, but the mental health impairments led to individuals performing too much physical activity. For example, a person with a high level of anxiety might have exercised obsessively because of psychopathological reasons. An intervention study is required in the future to investigated the causal associations and to determine the exact optimal levels of physical activity. Second, the participants of this study were recruited through acquaintances of people working at the university, and were neither randomized nor validated to represent the general population. Furthermore, the sample size of this study was not large enough to estimate the optimal levels of physical activity for different categories of the population, such as younger persons, older persons, persons with illnesses, and those without. It is expected that the optimal range will differ between age, gender, physical condition, etc. Therefore, a large-scale study would help to clarify the optimal range of physical activity for each group. Particularly in recent years, the circumstances of physical activity have dramatically changed all over the world due to the COVID-19 aftermath. The life and work environmental changes affect their mental health status including anxiety, depression, or insomniac symptoms (Wang et al., 2020; Shimura et al., 2021; Wang et al., 2021), however, our data was collected before the COVID-19 pandemic, and was hence not affected by it. Therefore, further studies are required to investigate the effect of the COVID-19 pandemic on the physical activity and mental health. Finally, physical activity was evaluated subjectively, and it may differ from objective physical activity.

### Conclusion

Physical activity in the optimal range was associated with more favorable mental health measurements, such as for depression, anxiety, resilience, and insomnia. The estimated optimal range of exercise volume was 5.3–9.2 k METs-minutes/week, and the optimal range of exercise duration was 21–31 h/week. Physical activities beyond or below the optimal ranges were associated with less favorable mental health measurements in the general adult population.

## Data availability statement

The raw data supporting the conclusions of this article will be made available by the authors, without undue reservation.

## Ethics statement

The studies involving human participants were reviewed and approved by the Tokyo Medical University Research Ethics Committee. The patients/participants provided their written informed consent to participate in this study.

## Author contributions

AS: conceptualization, methodology, formal analysis, investigation, and writing—original draft. JM: investigation, data curation, writing—review and editing, and supervision. KY: formal analysis and writing—original draft. CM, MK, and KN: investigation. CC and SN: methodology, validation, writing—review and editing, and supervision. TI: conceptualization, methodology, validation, resources, data curation, writing—review and editing, supervision, and project administration. All authors contributed to the article and approved the submitted version.

## Conflict of interest

The authors declare that the research was conducted in the absence of any commercial or financial relationships that could be construed as a potential conflict of interest.

## Publisher’s note

All claims expressed in this article are solely those of the authors and do not necessarily represent those of their affiliated organizations, or those of the publisher, the editors and the reviewers. Any product that may be evaluated in this article, or claim that may be made by its manufacturer, is not guaranteed or endorsed by the publisher.

## References

[ref1] Anderson T. Haake S. Lane A. R. Hackney A. C. (2016). Changes in resting salivary testosterone, cortisol and Interleukin-6 as biomarkers of overtraining. Balt. J. Sport Health Sci. 101, 2–7. PMID: 29708232 PMC5918265

[ref2] Armstrong L. E. VanHeest J. L. (2002). The unknown mechanism of the overtraining syndrome. Sports Med. 32, 185–209. doi: 10.2165/00007256-200232030-00003, PMID: 11839081

[ref3] Aylett E. Small N. Bower P. (2018). Exercise in the treatment of clinical anxiety in general practice–a systematic review and meta-analysis. BMC Health Serv. Res. 18, 1–18. doi: 10.1186/s12913-018-3313-5 30012142 PMC6048763

[ref4] Banno M. Harada Y. Taniguchi M. Tobita R. Tsujimoto H. Tsujimoto Y.. (2018). Exercise can improve sleep quality: a systematic review and meta-analysis. PeerJ 6:e5172. doi: 10.7717/peerj.5172, PMID: 30018855 PMC6045928

[ref5] Baron K. G. Reid K. J. Zee P. C. (2013). Exercise to improve sleep in insomnia: exploration of the bidirectional effects. J. Clin. Sleep Med. 9, 819–824. doi: 10.5664/jcsm.2930, PMID: 23946713 PMC3716674

[ref6] Bridle C. Spanjers K. Patel S. Atherton N. M. Lamb S. E. (2012). Effect of exercise on depression severity in older people: systematic review and meta-analysis of randomised controlled trials. Br. J. Psychiatry 201, 180–185. doi: 10.1192/bjp.bp.111.095174, PMID: 22945926

[ref7] Brown W. J. Ford J. H. Burton N. W. Marshall A. L. Dobson A. J. (2005). Prospective study of physical activity and depressive symptoms in middle-aged women. Am. J. Prev. Med. 29, 265–272. doi: 10.1016/j.amepre.2005.06.009 16242588

[ref8] Chekroud S. R. Gueorguieva R. Zheutlin A. B. Paulus M. Krumholz H. M. Krystal J. H.. (2018). Association between physical exercise and mental health in 1· 2 million individuals in the USA between 2011 and 2015: a cross-sectional study. Lancet Psychiatry 5, 739–746. doi: 10.1016/S2215-0366(18)30227-X, PMID: 30099000

[ref9] Childs E. de Wit H. (2014). Regular exercise is associated with emotional resilience to acute stress in healthy adults. Front. Physiol. 5:161. doi: 10.3389/fphys.2014.00161 24822048 PMC4013452

[ref10] Connor K. M. Davidson J. R. T. (2003). Development of a new resilience scale: the Connor-Davidson resilience scale (CD-RISC). Depress. Anxiety 18, 76–82. doi: 10.1002/da.10113 12964174

[ref11] Cortina J. M. (1993). What is coefficient alpha? An examination of theory and applications. J. Appl. Psychol. 78, 98–104. doi: 10.1037/0021-9010.78.1.98

[ref12] Craig C. L. Marshall A. L. Sjöström M. Bauman A. E. Booth M. L. Ainsworth B. E.. (2003). International physical activity questionnaire: 12-country reliability and validity. Med. Sci. Sports Exerc. 35, 1381–1395. doi: 10.1249/01.MSS.0000078924.61453.FB, PMID: 12900694

[ref13] de Zeeuw E. L. Tak E. C. Dusseldorp E. Hendriksen I. J. (2010). Workplace exercise intervention to prevent depression: a pilot randomized controlled trial. Ment. Health Phys. Act. 3, 72–77. doi: 10.1016/j.mhpa.2010.09.002

[ref14] Drake C. Richardson G. Roehrs T. Scofield H. Roth T. (2004). Vulnerability to stress-related sleep disturbance and hyperarousal. Sleep 27, 285–291. doi: 10.1093/sleep/27.2.285, PMID: 15124724

[ref15] Driver H. S. Taylor S. R. (2000). Exercise and sleep. Sleep Med. Rev. 4, 387–402. doi: 10.1053/smrv.2000.0110 12531177

[ref16] Dunn A. L. Trivedi M. H. Kampert J. B. Clark C. G. Chambliss H. O. (2005). Exercise treatment for depression: efficacy and dose response. Am. J. Prev. Med. 28, 1–8. doi: 10.1016/j.amepre.2004.09.003 15626549

[ref17] Dunn A. L. Trivedi M. H. O'Neal H. A.. (2001). Physical activity dose-response effects on outcomes of depression and anxiety. In Database of Abstracts of Reviews of Effects (DARE): Quality-assessed Reviews [Internet]. Centre for Reviews and Dissemination (UK). 10.1097/00005768-200106001-00027 11427783

[ref18] Hamer M. Stamatakis E. Steptoe A. (2009). Dose-response relationship between physical activity and mental health: the Scottish health survey. Br. J. Sports Med. 43, 1111–1114. doi: 10.1136/bjsm.2008.046243, PMID: 18403415

[ref19] Hansen C. J. Stevens L. C. Coast J. R. (2001). Exercise duration and mood state: how much is enough to feel better? Health Psychol. 20, 267–275. doi: 10.1037/0278-6133.20.4.267, PMID: 11515738

[ref20] Herring M. P. O’Connor P. J. Dishman R. K. (2010). The effect of exercise training on anxiety symptoms among patients: a systematic review. Arch. Intern. Med. 170, 321–331. doi: 10.1001/archinternmed.2009.530 20177034

[ref21] Hidano N Fukuhara M Iwawaki M Soga S Spielberger C. (2000). State-Trait Anxiety Inventory-Form Jyz. Tokyo: Japan UNI Agency (in Japanese).

[ref22] Jayakody K. Gunadasa S. Hosker C. (2014). Exercise for anxiety disorders: systematic review. Br. J. Sports Med. 48, 187–196. doi: 10.1136/bjsports-2012-091287 23299048

[ref23] Johnson M. B. Thiese S. M. (1992). A review of overtraining syndrome-recognizing the signs and symptoms. J. Athl. Train. 27, 352–354. PMID: 16558192 PMC1317287

[ref24] Josefsson T. Lindwall M. Archer T. (2014). Physical exercise intervention in depressive disorders: meta-analysis and systematic review. Scand. J. Med. Sci. Sports 24, 259–272. doi: 10.1111/sms.12050 23362828

[ref25] Kelley G. A. Kelley K. S. (2017). Exercise and sleep: a systematic review of previous meta-analyses. J. Evid. Based Med. 10, 26–36. doi: 10.1111/jebm.12236, PMID: 28276627 PMC5527334

[ref26] Kondo M. Shimada S. (2015). Serotonin and exercise-induced brain plasticity. Neurotransmitter:2. Available at: https://www.smartscitech.com/index.php/NT/article/view/871

[ref27] Kredlow M. A. Capozzoli M. C. Hearon B. A. Calkins A. W. Otto M. W. (2015). The effects of physical activity on sleep: a meta-analytic review. J. Behav. Med. 38, 427–449. doi: 10.1007/s10865-015-9617-6, PMID: 25596964

[ref28] Kroenke K. Spitzer R. L. Williams J. B. (2001). The Phq-9: validity of a brief depression severity measure. J. Gen. Intern. Med. 16, 606–613. doi: 10.1046/j.1525-1497.2001.016009606.x, PMID: 11556941 PMC1495268

[ref29] Krogh J. Nordentoft M. Sterne J. A. Lawlor D. A. (2010). The effect of exercise in clinically depressed adults: systematic review and meta-analysis of randomized controlled trials. J. Clin. Psychiatry 71:5500. doi: 10.4088/JCP.08r04913blu 21034688

[ref30] Maddock R. J. Casazza G. A. Fernandez D. H. Maddock M. I. (2016). Acute modulation of cortical glutamate and GABA content by physical activity. J. Neurosci. 36, 2449–2457. doi: 10.1523/jneurosci.3455-15.2016, PMID: 26911692 PMC6705493

[ref31] Marcos de Souza Moura A. Khede Lamego M. Paes F. Ferreira Rocha N. B. Simoes-Silva V. Almeida Rocha S.. (2015). Effects of aerobic exercise on anxiety disorders: a systematic review. CNS Neurol. Disord. Drug Targets 14, 1184–1193. doi: 10.2174/1871527315666151111121259 26556089

[ref32] Miyama H. Shimura A. Furuichi W. Seki T. Ono K. Masuya J.. (2020). Association of chronotypes and sleep disturbance with perceived job stressors and stress response: a covariance structure analysis. Neuropsychiatr. Dis. Treat. 16, 1997–2005. doi: 10.2147/NDT.S262510, PMID: 32904619 PMC7457392

[ref33] Morse C. D. Klingman K. J. Jacob B. L. Kodali L. (2019). Exercise and insomnia risk in middle-aged women. J. Nurse Pract. 15, 236–240.e2. doi: 10.1016/j.nurpra.2018.10.020

[ref34] Muramatsu K. Kamijima K. Yoshida M. Otsubo T. Miyaoka H. Muramatsu Y.. (2007). The patient health questionnaire, Japanese version: validity according to the mini-international neuropsychiatric interview–plus. Psychol. Rep. 101, 952–960. doi: 10.2466/pr0.101.3.952-960 18232454

[ref35] Murase N. (2002). Validity and reliability of Japanese version of international physical activity questionnaire. J. Health Welfare Stat. 49, 1–9.

[ref36] Nakai Y. Inoue T. Toda H. Toyomaki A. Nakato Y. Nakagawa S.. (2014). The influence of childhood abuse, adult stressful life events and temperaments on depressive symptoms in the nonclinical general adult population. J. Affect. Disord. 158, 101–107. doi: 10.1016/j.jad.2014.02.004, PMID: 24655773

[ref38] Nakajima S. Okajima I. Sasai T. Kobayashi M. Furudate N. Drake C. L.. (2014). Validation of the Japanese version of the ford insomnia response to stress test and the association of sleep reactivity with trait anxiety and insomnia. Sleep Med. 15, 196–202. doi: 10.1016/j.sleep.2013.09.022, PMID: 24380783

[ref39] Nebiker L. Lichtenstein E. Minghetti A. Zahner L. Gerber M. Faude O.. (2018). Moderating effects of exercise duration and intensity in neuromuscular Vs. endurance exercise interventions for the treatment of depression: a meta-analytical review. Front. Psych. 9:305. doi: 10.3389/fpsyt.2018.00305 PMC6060256 30072923

[ref37] Nishi D. Uehara R. Kondo M. Matsuoka Y. (2010). Reliability and validity of the Japanese version of the Resilience Scale and its short version. BMC Res Notes. 3, 1–6. doi: 10.1186/1756-0500-3-310 21083895 PMC2993730

[ref40] Petruzzello S. J. Landers D. M. Hatfield B. D. Kubitz K. A. Salazar W. (1991). A meta-analysis on the anxiety-reducing effects of acute and chronic exercise. Sports Med. 11, 143–182. doi: 10.2165/00007256-199111030-00002, PMID: 1828608

[ref41] Sameshima H. Shimura A. Ono K. Masuya J. Ichiki M. Nakajima S.. (2020). Combined effects of parenting in childhood and resilience on work stress in nonclinical adult workers from the community. Front. Psych. 11:776. doi: 10.3389/fpsyt.2020.00776, PMID: 32848942 PMC7411223

[ref42] Sarason I. G. Johnson J. H. Siegel J. M. (1978). Assessing the impact of life changes: development of the life experiences survey. J. Consult. Clin. Psychol. 46, 932–946. doi: 10.1037/0022-006X.46.5.932 701572

[ref43] Schuch F. B. Vancampfort D. Richards J. Rosenbaum S. Ward P. B. Stubbs B. (2016). Exercise as a treatment for depression: a meta-analysis adjusting for publication bias. J. Psychiatr. Res. 77, 42–51. doi: 10.1016/j.jpsychires.2016.02.023, PMID: 26978184

[ref44] Shimura A. Yokoi K. Ishibashi Y. Akatsuka Y. Inoue T. (2021). Remote work decreases psychological and physical stress responses, but full-remote work increases presenteeism. Front. Psychol. 12:730969. doi: 10.3389/fpsyg.2021.730969, PMID: 34659039 PMC8514617

[ref45] Smith L. L. (2000). Cytokine hypothesis of overtraining: a physiological adaptation to excessive stress. Med. Sci. Sports Exerc. 32, 317–331. doi: 10.1097/00005768-200002000-00011, PMID: 10694113

[ref46] Spielberger C. D. Gonzalez-Reigosa F. Martinez-Urrutia A. Natalicio L. F. Natalicio D. S. (1971). The state-trait anxiety inventory. Rev. Int. Psicol. 5:730969.

[ref47] Stonerock G. L. Hoffman B. M. Smith P. J. Blumenthal J. A. (2015). Exercise as treatment for anxiety: systematic review and analysis. Ann. Behav. Med. 49, 542–556. doi: 10.1007/s12160-014-9685-9, PMID: 25697132 PMC4498975

[ref48] Szuhany K. L. Bugatti M. Otto M. W. (2015). A meta-analytic review of the effects of exercise on brain-derived neurotrophic factor. J. Psychiatr. Res. 60, 56–64. doi: 10.1016/j.jpsychires.2014.10.003 25455510 PMC4314337

[ref49] Teychenne M. Ball K. Salmon J. (2008). Physical activity and likelihood of depression in adults: a review. Prev. Med. 46, 397–411. doi: 10.1016/j.ypmed.2008.01.009 18289655

[ref50] Tokunaga K. Matsuzawa Y. Kotani K. Keno Y. Kobatake T. Fujioka S.. (1991). Ideal body weight estimated from the body mass index with the lowest morbidity. Int. J. Obes. 15, 1–5. PMID: 2010254

[ref51] Wang S. Zhang Y. Ding W. Meng Y. Hu H. Liu Z.. (2020). Psychological distress and sleep problems when people are under interpersonal isolation during an epidemic: a nationwide multicenter cross-sectional study. Eur. Psychiatry 63:e77. doi: 10.1192/j.eurpsy.2020.78, PMID: 32854786 PMC7503168

[ref52] Wang S. Zhang Y. Guan Y. Ding W. Meng Y. Hu H.. (2021). A nationwide evaluation of the prevalence of and risk factors associated with anxiety, depression and insomnia symptoms during the return-to-work period of coronavirus disease 2019 in China. Soc. Psychiatry Psychiatr. Epidemiol. 56, 2275–2286. doi: 10.1007/s00127-021-02046-4, PMID: 33616693 PMC7898251

[ref53] Wegner M. Helmich I. Machado S. E. Nardi A. Arias-Carrion O. Budde H. (2014). Effects of exercise on anxiety and depression disorders: review of meta- analyses and neurobiological mechanisms. Curr. Drug Targets CNS Neurol. Disord. 13, 1002–1014. doi: 10.2174/1871527313666140612102841, PMID: 24923346

[ref54] Wyatt F. B. Donaldson A. Brown E. (2013). The overtraining syndrome: a meta-analytic review. J. Exerc. Physiol. 16, 12–23.

